# The Development of a Stable Peptide-Loaded Long-Acting Injection Formulation through a Comprehensive Understanding of Peptide Degradation Mechanisms: A QbD-Based Approach

**DOI:** 10.3390/pharmaceutics16020266

**Published:** 2024-02-13

**Authors:** Yingxin Xiong, Jiawei Wang, Xing Zhou, Xiaohui Li

**Affiliations:** 1 Institute of Materia Medica and Department of Pharmaceutics, College of Pharmacy, Army Medical University, Chongqing 400038, China; yx.xiong@gmail.com; 2 Chongqing School of Pharmacy and Bioengineering, Chongqing University of Technology, Chongqing 400054, China; wangjiawei0423@gmail.com; 3 Engineering Research Center for Pharmacodynamics Evaluation, College of Pharmacy, Army Medical University, Chongqing 400038, China

**Keywords:** quality by design (QbD), control strategy, peptide, stability, degradation mechanisms, long-acting injection

## Abstract

Quality by design (QbD) serves as a systematic approach to pharmaceutical development, beginning with predefined objectives and emphasizing an understanding of the product based on sound science and risk management. The purpose of this study is to utilize the QbD concept to develop a stable peptide-loaded long-acting injection formulation. An in-depth comprehension of peptide degradation mechanisms was achieved through forced degradation investigations, elucidating (acid) hydrolysis and oxidation as the primary degradation pathways for the peptide ACTY116. The quality built into the product was focused on risk assessment, for which the critical material attributes (CMAs) and critical process parameters (CPPs) associated with the critical quality attributes (CQAs) of each formulation were identified, leading to the development of the corresponding control strategies. CQAs for three LAI (long-acting injectable) formulations were enhanced by taking the right control strategies. The LAI formulation exhibiting the highest stability for ACTY116 was chosen for subsequent pharmacokinetic investigations in rats. The objective of addressing peptide chemical instability and in vivo long-acting release was achieved. For other molecules with susceptible functionalities like amide bonds, amino groups, and hydroxyl groups, the utilization of PLGA-based in situ gel as an LAI formulation for stabilizing molecules provides valuable insights.

## 1. Introduction

**Figure 1:**
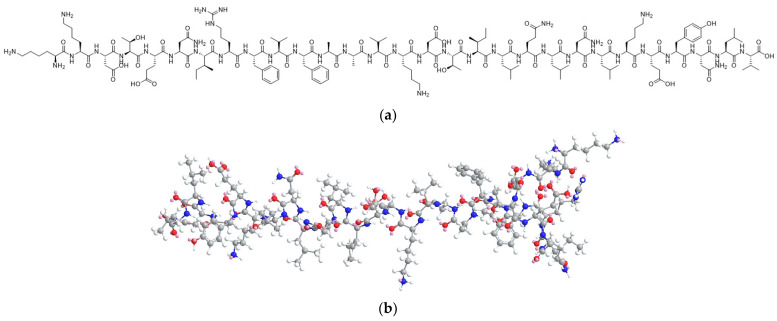
The structure of peptide ACTY116: (**a**) 2D, (**b**) 3D.

In recent years, the field of peptide therapeutics has undergone substantial advancements. These therapeutic peptides offer several advantages over biologics, including reduced immunogenicity and enhanced cost-effectiveness. Moreover, they exhibit superior safety, selectivity, efficacy, and specificity when compared to small-molecule drugs. One such peptide, ACTY116 (the structure is shown in Figure 1), comprising 29 amino acids (C_157_H_256_N_40_O_45_, with a molecular weight of 3424 g/mol), was designed in our laboratory as a competitive ligand for the binding sites on Gαq (the activated α subunit of the heterotrimeric G protein) in cardiomyocytes. Extensive investigations have been undertaken concerning the structural design of the peptide and the antihypertrophic evaluation of both GCIP (Gαq protein carboxyl terminus imitation peptide) and ACTY116 (an analog of GCIP with specific structural modification) [1,2,3]. These studies have shown that ACTY116 holds considerable promise as a potential drug candidate for advancing into clinical development. Despite these findings, the formulation design required for its clinical application has yet to be explored.

Similar to other peptides, ACTY116 presents a set of challenges and limitations that require careful consideration for their successful translation into clinically viable therapeutic products. Peptides typically exhibit a short half-life due to rapid renal filtration and enzymatic degradation. As a result, maintaining a therapeutic level often demands frequent injections or continuous infusions, potentially leading to issues of patient compliance and escalating treatment expenses. Additionally, ACTY116 is susceptible to oxidation and hydrolysis due to the presence of numerous amide bonds, phenolic hydroxyl groups, and amino groups. This chemical instability could compromise both its effectiveness and safety [4,5,6,7]. Simultaneously addressing peptide chemical instability and achieving long-acting release presents a substantial challenge.

To address the challenge of limited drug half-life, the utilization of long-acting injectables (LAIs) has emerged as a viable strategy, providing extended drug release through various approaches: (1) chemical modification [8]: modifying drug molecules (such as semaglutide) can enhance stability and prolong drug release [9,10]; (2) active pharmaceutical ingredients (APIs) with low solubility, like Invega Hafyera [11,12], form suspensions for gradual release. The hydrophilic nature of ACTY116 makes it unsuitable for this approach; (3) nonbiodegradable implants (such as Viadur) deliver the drug over months or even years but pose challenges to patient compliance due to the necessity of surgical removal [13]; (4) the formation of drug depots or reservoirs using a biodegradable matrix: The drug is typically formulated as a solution or suspension that undergoes slow release from the depot, extending the availability of the drug. The drug-loaded biodegradable microspheres or in situ gels are administered via injection, and over time, the polymer breaks down into biocompatible byproducts, eliminating the need for device removal [14,15,16,17,18,19].

Biodegradable microspheres and in situ gels were chosen as LAI approaches for investigating ACTY116, with PLGA and soybean phospholipid selected as the long-acting matrix materials. PLGA (poly(D,L-lactide-co-glycolide)) has achieved success in various US and EU commercial products, including Lupron Depot (leuprolide acetate), Trelstar (triptorelin pamoate), and Risperdal Consta (risperidone) [20,21,22,23,24]. It undergoes biodegradation in vivo, producing biocompatible byproducts like lactic and glycolic acids that can be cleared via the Krebs cycle. The release kinetics of the active pharmaceutical ingredient (API) from PLGA primarily depend on drug diffusion and polymer erosion/degradation process [25]. Soybean phospholipid, comprising a glycerol backbone, two fatty acid chains, a phosphate group, and a choline head group, possesses amphiphilic properties. These properties make it a versatile emulsifier and stabilizer of oil–water interfaces. When employed as a long-acting matrix within in situ gels, soybean phospholipid forms a cross-linked gel network upon administration. This network acts as a barrier, hindering rapid drug release. As the phospholipid matrix gradually degrades over time, the drug is released in a controlled manner, resulting in an extended-release profile [26,27,28,29].

**Figure 2:**
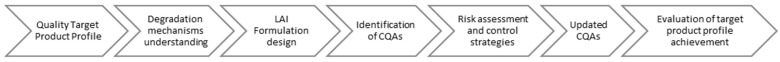
Schematic illustration of long-acting injection development based on quality by design (QbD).

The objective of this study was to develop a stable peptide-loaded long-acting injection by following QbD principles (Figure 2). We conducted our study by adhering to the ICH guidelines (Q8, Q9, and Q10) and incorporating recommendations from a highly cited review article titled “Understanding Pharmaceutical Quality by Design” on how to carry out pharmaceutical product development based on the QbD concept [30]. A quality target product profile (QTPP) was established, and ACTY116-loaded LAI formulations were designed based on a comprehensive understanding of ACTY116 degradation mechanisms. The quality built into the product focused on risk assessment, identifying critical material attributes (CMAs) and critical process parameters (CPPs) associated with the critical quality attributes (CQAs) of each formulation, leading to the development of corresponding control strategies. CQAs for different LAI formulations were enhanced by taking the right control strategies. The LAI formulation exhibiting the highest stability for ACTY116 was chosen for further pharmacokinetic investigations in rats. The results were compared with the target product profile to assess the achievement of stability and in vivo long-acting pharmacokinetic behavior objectives.

## 2. Materials and Methods

### 2.1. Materials

ACTY116 was synthesized by a contract research organization (HLXK, Beijing, China), Poly (D,L-lactide-co-glycolide) (PLGA, with a ratio of lactide to glycolide at 50:50, MW:7000–17,000, acid-terminated) was purchased from Evonik (Darmstadt, Germany), N-methylpyrrolidone (NMP) and dichloromethane (DCM) were purchased from Chengdu Kelong Chemical Co., Ltd. (Chengdu, China), mannitol was purchased from Roquette (Lestrem, France), phospholipid was purchased from Lipoid (Ludwigshafen, Germany), medium chain triglycerides (MCTs) were purchased from Shinsun pharma (Tieling, China), and p-anisidine was purchased from Sinopharm (Shanghai, China).

### 2.2. Equipment and Instruments

The instruments used in this study included a high-performance liquid chromatograph (e2695-2998, Waters, Milford, MA, USA), a digital mixer (RW20, IKA, Staufen, Germany), a lyophilizer (D-37520, Christ, Osterode, Germany), a polarizing microscope (BK-POL, Aote Optical Instrument, Chongqing, China), a thermostatic oscillator (SHA-C, GY2016-SW, Changzhou Guoyu, Changzhou, China), a digital rotary viscometer (NDJ-8S, Fangrui, Shanghai, China), laser-diffraction particle size analyzers (Mastersizer 3000, Malvern, Worcestershire, UK), a moisture analyzer (Metrohm, Herisau, Switzerland), heat and accelerated stability chambers (Yongsheng, Chongqing, China), a UV spectrophotometer (UV 2450, Shimadzu, Kyoto, Japan), an ultra-high-performance liquid chromatograph (e2695-2998, Waters, Milford, MA, USA), an LC-MS/MS system (6460, Agilent, Santa Clara, CA, USA), and a refrigerated centrifuge (Legend Micro 17R, Thermo, Waltham, MA, USA).

### 2.3. Degradation Mechanism Studies of ACTY116

#### 2.3.1. Method for ACTY116 Impurity Analysis

The analytical method should be capable of capturing all the potential degradation impurities formed during the stability study. High-performance liquid chromatography (HPLC) was used as the stability-indicating method for ACTY116 impurity analysis; the detailed information is described below:

- Chromatographic column: XSelect CSH C18, 150 × 4.6 mm, 5 μm;
- Wavelength: 210 nm;
- Column temperature: 40 °C;
- Flow rate: 1 mL/min;
- Injection volume: 25 μL;
- Mobile phase:
  ○ A: Using phosphate buffer pH 6.5 (containing 20 mM sodium perchlorate);
  ○ B: Using acetonitrile–water (*v*/*v*) = 80:20.

Gradient elution is shown in Table 1:

#### 2.3.2. Forced Degradation Studies

In order to comprehend the chemical properties of ACTY116, a set of stress conditions was utilized to expedite its chemical degradation. This approach enabled the comprehensive assessment of its intrinsic stability and the delineation of degradation pathways [31,32,33]. The specific studies are outlined as follows:

a. Thermal stress: A measured quantity of ACTY116 was placed into a lidded glass vial and subjected to thermal stress at 60 °C for a duration of 5 days in a heat chamber. Subsequent to the stress period, 1 mg of ACTY116 was precisely weighed and dissolved in 1 mL of purified water for HPLC analysis.

b. Photolytic stress: A measured quantity of ACTY116 was placed into a lidded glass vial and exposed to light under 4500 Lux for a duration of 5 days; then, 1 mg of ACTY116 was precisely weighed and dissolved in 1 mL of purified water for HPLC analysis.

c. Oxidative stress: A measured quantity of ACTY116 (2 mg) was placed into a glass vial and dissolved in 2 mL of 3% H_2_O_2_. The solution was then subjected to incubation in an oil bath at 100 °C for a duration of 2 h, followed by cooling to room temperature prior to subsequent HPLC analysis.

d. Hydrolytic stress: A measured quantity of ACTY116 (2 mg) was placed into a glass vial and dissolved in 2 mL of purified water. The solution was then subjected to incubation in an oil bath at 100 °C for a duration of 2 h, followed by cooling to room temperature prior to HPLC analysis.

e. Acidic hydrolytic stress: A measured quantity of ACTY116 (2 mg) was placed into a glass vial and dissolved in 2 mL of 1M HCl. The solution was then subjected to incubation in an oil bath at 100 °C for a duration of 2 h, followed by cooling to room temperature prior to HPLC analysis.

### 2.4. LAI Formulation Design

Three different LAI formulations were designed in this study, and the ingredients in each formulation are presented in Table 2.

#### 2.4.1. Preparation of Microspheres

**Figure 3:**
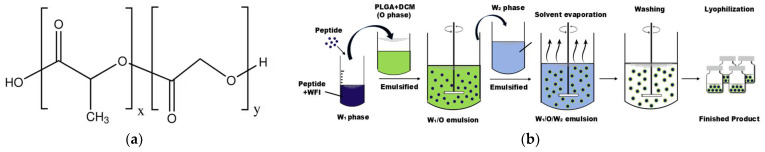
(**a**) PLGA structure; (**b**) schematic illustration of double emulsion-solvent extraction/evaporation technique for microsphere preparation.

The double emulsion-solvent extraction/evaporation method (Figure 3b) was adopted due to the high hydrophilicity of ACTY116. ACTY116 (2 mg) was weighed into a glass vial and dissolved in 200 μL of water for injection (WFI) as the water phase (W_1_ phase), and 300 mg of PLGA (with a ratio of lactide to glycolide at 50:50, MW:7000–17,000, acid-terminated, Figure 3a) was dissolved in 2 mL of DCM as the oil phase. A hydrophilic drug solution (W_1_ phase) was emulsified in an organic polymer solution (O phase) under 15,000 rpm high-shear mixing to form the primary water-in-oil (W_1_/O) emulsion. The obtained W_1_/O emulsion was subsequently added to 200 mL of 1% PVA solution (W_2_ phase) under 25,000 rpm high-shear mixing to form a double emulsion (W_1_/O/W_2_). The evaporation of DCM was performed under 50 ± 1 °C, 100 rpm continuous shaking for 4 h. The microsphere suspension was centrifuged at 5000 rpm for 2 min (4 °C) and then dispersed with 10 mL of WFI for washing the unencapsulated ACTY116. The washing step was repeated for 5 cycles. Finally, the microspheres were dispersed in a 10% mannitol solution, filled into vials, and lyophilized (F1) with the process shown below (Table 3).

#### 2.4.2. Preparation of Phospholipid-Based In Situ Gel

**Figure 4:**
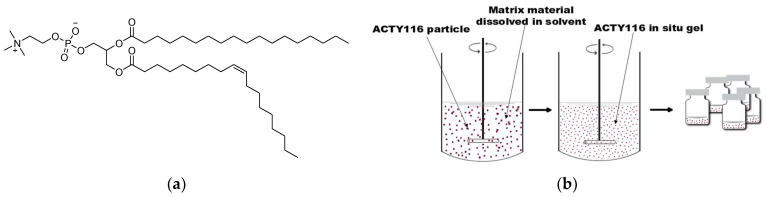
(**a**) Phospholipid structure; (**b**) schematic illustration of in situ gel preparation.

Soybean phospholipid (Figure 4a) was selected as the matrix for the in situ gel formulation (F2). Briefly, 1.5 g of soybean phospholipid was dissolved in 3.0 g of medium chain triglycerides (MCTs) under stirring at 80 °C. The mixture was subsequently cooled to room temperature, and then 50 mg of ACTY116 was introduced and stirred for approximately 10 min until the formation of a visible suspension. This resulting suspension was then filled into vials for subsequent use. The preparation method is illustrated in Figure 4b.

#### 2.4.3. Preparation of PLGA-Based In Situ Gel

PLGA (with a ratio of lactide to glycolide at 50:50, MW:7000–17,000, acid-terminated) was selected as the polymer to serve as the foundation for the long-acting matrix. The process involved the initial dissolution of 660 mg of PLGA into 1280 mg of NMP; subsequently, 56 mg of ACTY116 was introduced into the solution and stirred for approximately 10 min until the formation of a visibly apparent suspension. This resulting suspension was then transferred into vials for subsequent use (F3).

#### 2.4.4. Characterization of Different Formulations

Polarized microscopic examination was performed by dispersing ACTY116 microspheres in 10 mL of purified water, forming a suspension that was then dropped onto a glass slide. The observation process involved the following parameters: an exposure time of 350 ms, a polarization angle of 4°, and a magnification factor of 30×. The same set of parameter settings was used to examine the in situ gel suspensions after they were applied to glass slides [34,35].

Particle size determination: Particle sizes were determined by employing laser diffraction particle size analyzers. The lyophilized microsphere cake was reconstituted with 2 mL of purified water to form a suspension, which was subsequently dispersed in 250 mL of purified water. Instrument settings included a background measurement duration of 10 s, a sample measurement duration of 30 s, an obscuration range set between 5.0% and 10.0%, and a stirrer speed of 1000 rpm. Similarly, 1 mL of phospholipid-based in situ gel was dispersed in 250 mL of MCT, and 1 mL of PLGA-based in situ gel was dispersed in 250 mL of NMP, with particle size determined using the same parameters.

Encapsulation efficiency (EE): Briefly, 40 mg of microspheres was added to a 5 mL centrifuge tube. Subsequently, 4 mL of purified water was added, and the mixture was shaken to ensure the uniform dispersion of the microspheres. After centrifugation at 10,000 rpm for 5 min, the supernatant was collected to measure unencapsulated peptides. The microspheres settled at the bottom were transferred to a 25 mL volumetric flask using DMSO, the centrifuge tube was rinsed with DMSO five times, and the rinses were pooled in the same volumetric flask. The mixture was then diluted to volume and thoroughly mixed, and the encapsulated peptide (ACTY116) was quantified using HPLC. Encapsulation efficiency was calculated using the following expression:

(1) $$\mathrm{Encapsulation}\;\mathrm{efficiency}\;\left(\mathrm{EE}\right)\%=\frac{\mathrm{Encapsulated}\;\mathrm{peptide}}{\mathrm{Unencapsulated}\;\mathrm{peptide}+\mathrm{Encapsulated}\;\mathrm{peptide}}\times 100\%$$

In situ gel viscosity was assessed by using a digital rotary viscometer. The suspension was put in the rotor, after which the instrument was started with a parameter configuration of 3.0 rpm for a duration of 2 min [36]. Then, the viscosity result displayed on the screen after the measurement was recorded.

### 2.5. Identification of CQAs

#### 2.5.1. Impurity Profiles (CQA1) for Different Formulations

Considering the chemical instability of the peptide ACTY116, impurity profiles were identified as critical quality attributes (CQA1). In order to assess the impurity profiles of ACTY116 across different formulations, total impurities were analyzed following storage in a stability chamber (40 °C, 75% RH) for 10 and 20 days.

#### 2.5.2. Other Critical Quality Attributes (CQA2) for Different Formulations

This study primarily focused on addressing the issue of peptide instability, and therefore the in vitro release was not included as a CQA in this study even though it is important to LAI formulations.

Given that (acid) hydrolysis and oxidation were degradation paths of ACTY116, water content, acid value, iodine value, peroxide value, and AV were also considered critical quality attributes (CQA2) for different formulations in this study.

Water content measurement: The sample was weighed and dissolved in anhydrous methanol, and the water content was determined utilizing a moisture analyzer.

The acid value I_A_ is the number that expresses, in milligrams, the quantity of potassium hydroxide required to neutralize the free acids present in 1 g of the samples. The iodine value I_I_ is the number that expresses, in grams, the quantity of halogen, calculated as iodine, which can be fixed in the prescribed conditions by 100 g of the sample. The testing methods of I_A_ and I_I_ follow general monograph 2.5.4 in the European Pharmacopoeia [37]. The peroxide value I_P_ is defined as the quantity of peroxide contained in 1000 g of the substance and expressed as in milliequivalents of active oxygen. The testing method follows general monograph 2.5.5 in the European Pharmacopoeia. The anisidine value (AV) is used to evaluate the quantity of aldehydes and ketones in pharmaceutical products. It serves as an important indicator of the extent of the oxidative degradation of lipids in products. AV determination is based on the reaction of aldehydes and ketones with anisidine under alkaline conditions. Aldehydes and ketones undergo a Schiff base reaction (nucleophilic addition) with anisidine under acidic catalysis. The carbonyl group reacts with the amino group of anisidine to form an unstable intermediate (aldimine) structure; then, intramolecular dehydration occurs, yielding a stable colored product, imine. The absorption was measured by UV spectrophotometry to evaluate the quantity of aldehydes and ketones in the sample [38,39]. The testing method follows general monograph 2.5.36 in the European Pharmacopoeia.

### 2.6. Risk Assessment and Control Strategies

A fundamental aspect of pharmaceutical drug development involves identifying and controlling the critical material attributes (CMAs) and critical process parameters (CPPs) that influence critical quality attributes (CQAs). In this study, we identified CMAs for the input materials (excipients) by understanding the degradation mechanisms of ACTY116. Additionally, we recognized certain process parameters that can directly or indirectly impact peptide stability, particularly those related to peptide oxidation or hydrolysis, such as high temperature and oxygen exposure, as CPPs. To ensure quality is embedded into the product, we focused on risk assessment, which involved the identification of CMAs and CPPs associated with the critical quality attributes (CQAs) for each formulation, ultimately leading to the development of corresponding control strategies.

### 2.7. Updated CQAs for Different Formulations

An assessment of updated CQAs for different formulations was conducted following the implementation of control strategies. The updated impurity profile (CQA1) for each formulation was investigated by following their storage in an accelerated stability chamber (40 °C, 75% RH) for 10 and 20 days. The other updated critical quality attributes (CQA2) for different formulations such as the water content, acid value, iodine value, peroxide value, and AV were also analyzed.

### 2.8. Pharmacokinetic Study in Rats

#### 2.8.1. LC-MS/MS Method

The plasma concentrations of ACTY116 were analyzed using an LC-MS/MS system, which consisted of an Agilent 1290 Infinity II UHPLC system coupled with an Agilent 6460 triple quadrupole mass spectrometer equipped with an electrospray ionization source (ESI). The Agilent MassHunter workstation qualitative analysis B.08.00 (Agilent Technologies, Santa Clara, CA, USA) software was used. Chromatographic separation was performed on a BEH C18 column (2.1 mm × 75 mm, 1.7 µm, Waters, Milford, CT, USA). The separation conditions were 40 °C by using formic acid (FA)–water (0.1:99.9, *v*/*v*, A) and FA–acetonitrile (CAN) (0.1:99.9, *v*/*v*, B) solutions at a flow rate of 0.4 mL/min. The sample injection volume was 10 µL, and the autosampler was kept at 4 °C. The initial eluent AB (80:20, *v*/*v*) was changed to A-B (40:60, *v*/*v*) in 6 min and subsequently changed to A-B (20:80, *v*/*v*) in the next 0.5 min in a liner gradient and maintained at this ratio for another 1.5 min. Leuprolide acetate was used as the internal standard (IS). MS analysis was carried out in the multiple-reaction monitoring (MRM) mode with the following operation parameters: capillary voltage, 4.5 KV; gas temperature, 350 °C; nebulizer gas pressure, 50 psi; sheath gas, 11 L/min, 300 °C; fragmentor, 120 V for ACTY116 and IS; collision energy (CE), 15 eV for ACTY116 and 35 eV for IS; and precursor-to-product ion transition, *m*/*z*: 685.8→118.1 for ACTY116 and *m*/*z*: 606.5→249.4 for IS. Pharmacokinetic parameters were calculated using Phoenix 64 (version 8.3.3.33), and the noncompartmental analysis of pharmacokinetic data was conducted with the ‘linear-up log-down’ method.

#### 2.8.2. PK Study in Rats

SD rats (200–250 g) were purchased from Ensiweier Biotechnology, Chongqing, China. All animals had free access to a standard diet and drinking water and were housed in a room maintained at 22.0 ± 3 °C and with a 12:12 h cyclic lighting schedule. All animal experiments were approved by the Laboratory Animal Welfare and Ethics Committee of Army Medical University (approval no.: AMUWEC20203377), and all of the experiments were performed in accordance with the National Institutes of Health guidelines for the care and use of laboratory animals.

ACTY116 solution was injected subcutaneously in rats for the analysis of pharmacokinetic (PK) behavior as an immediate-release formulation. Six (6) rats received the ACTY116 solution at a dose of 1.0 mg/kg, and blood samples were collected from the retrobulbar venous plexus 10, 20, 40, 60, 90, 120, and 180 min after subcutaneous administration.

The most stable LAI formulation (PLGA-based in situ gel) was chosen for further pharmacokinetic investigations in rats. Six (6) rats received ACTY116 LAI formulation at a dose of 7 mg/kg. The blood samples were collected from the retrobulbar venous plexus 1, 2, and 4 h post-subcutaneous injection, on days 1, 3, 5, 7, 9, 11, 13, 15, and 17 (i.e., blood was collected every two days).

Plasma was separated via refrigerated centrifugation, and ACTY116 was analyzed with the LC-MS/MS method.

### 2.9. Evaluation of Target Product Profile Achievement

By comparing the results with the target product profile, the achievement of stability and in vivo long-acting pharmacokinetic behavior objectives was evaluated.

## 3. Results

### 3.1. Quality Target Product Profile

This study primarily addresses two objectives: firstly, to design and optimize long-acting injectable (LAI) formulations for maintaining peptide stability during storage and in vivo release, and secondly, to ensure that the LAI formulation can achieve long-acting pharmacokinetic (PK) behavior in rats for at least one week.

The in vivo long-acting characteristic was attained through formulation design using a biodegradable matrix. Consequently, the formulation development based on QbD primarily focused on addressing stability issues in this research.

The quality target product profile was established by referencing the total impurities limits specified in the United States Pharmacopeia (USP) for peptide injectable products, such as exenatide injection (≤10.0%) and teriparatide injection (≤7.0%), both stored at 2–8 °C. We established a target product impurity profile for ACTY116-loaded LAI formulation: In the preparation process, the growth of total impurities should not exceed 1.0%; for the finished product, an accelerated stability study should be undertaken at 40 °C, and the total impurities’ growth should not exceed 3.5% in 10 days and 7.0% in 20 days.

### 3.2. Degradation Mechanism Studies on ACTY116

**Figure 5:**
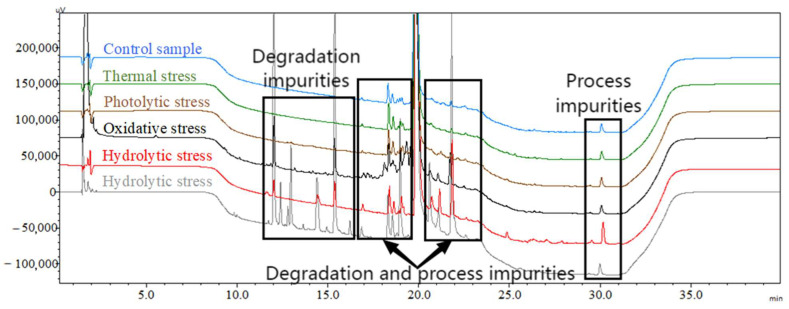
The typical HPLC chromatograms of ACTY116 under various forced degradation conditions; the *X*-axis represents the time, and the *Y*-axis represents the signal intensity: blue—control sample (nonstressed); green—thermal stress; brown—photolytic stress; black—oxidative stress; red—hydrolytic stress; gray—hydrolytic stress under acidic conditions.

The HPLC chromatograms in Figure 5 depict the impurity profiles resulting from different forced degradation conditions, illustrating variations in impurity levels.

The results demonstrated that ACTY116 (with its main peak observed at a retention time of approximately 20 min) remained stable under thermal and photolytic stress conditions, as evidenced by the absence of new degradation impurities compared to the control sample (nonstressed). However, oxidative degradation and (acid) hydrolysis led to a series of new degradation impurities, emphasizing these as the primary degradation pathways for the peptide ACTY116. Notably, hydrolytic stress under acidic conditions generated particularly pronounced degradation impurities, surpassing those observed in hydrolytic and oxidative stress. The total impurities are presented in Table 4.

The ACTY116 structure (Figure 1) shows the presence of 32 amide bonds and 1 guanidine group, which account for its susceptibility to hydrolysis. Additionally, the presence of one phenolic hydroxyl group, five amino groups, and two hydroxyl groups contributes to its susceptibility to oxidation. The forced degradation results align with the predicted chemical instability of the peptide structure. The degradation and process impurities are shown on the HPLC chromatogram.

### 3.3. Characterization of Different LAI Formulations

**Figure 6:**
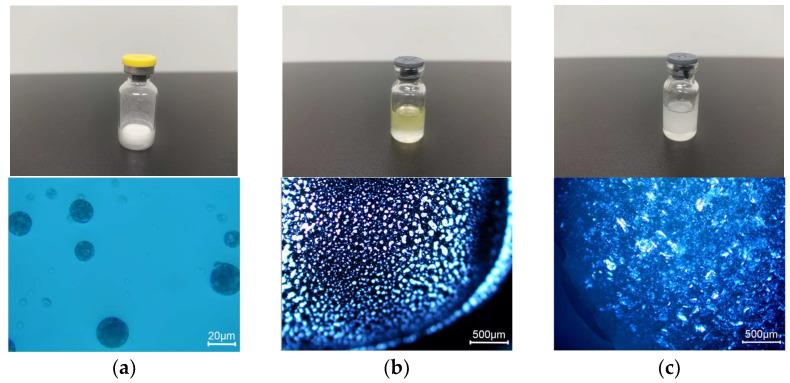
Visual appearance and polarizing microscope observation of three formulations: (**a**) microspheres (F1); (**b**) phospholipid-based in situ gel (F2); (**c**) PLGA-based in situ gel (F3).

Figure 6 presents the visual appearance and results of polarizing microscopic observation.

Three formulations are all in the form of suspension, and their particle size, encapsulation efficiency, and viscosity are listed in Table 5.

### 3.4. Initial CQAs

#### 3.4.1. Initial Impurity Profiles (CQA1) for Different Formulations

The chromatograms indicate that the peaks appearing within the retention time range of 24.0 min to 30.0 min are associated with the excipients. In the impurity analysis conducted during the stability study, the subtraction of these impurities was taken into account.

**Figure 7:**
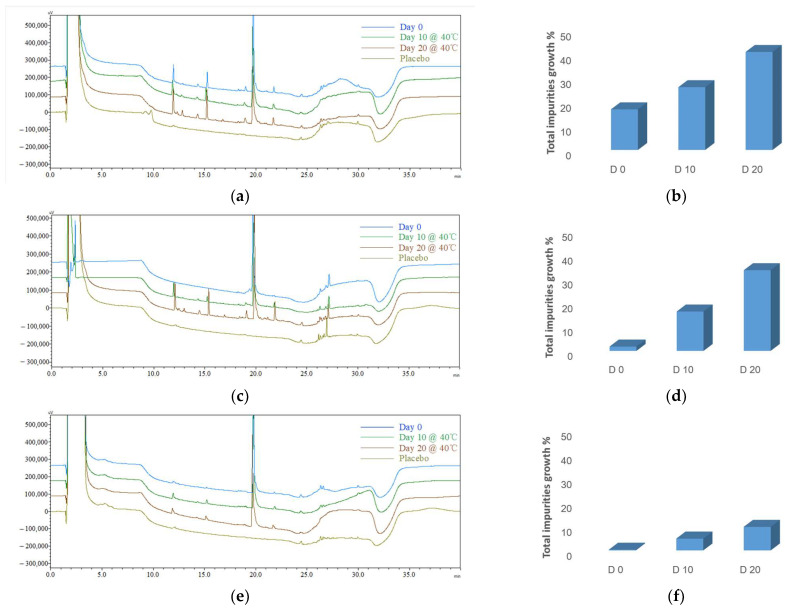
The typical HPLC chromatograms (left: **a**,**c**,**e**; the *X*-axis represents the time, and the *Y*-axis represents the signal intensity) and the impurity profile charts (right: **b**,**d**,**f**) for different formulations. The typical HPLC chromatograms (left: **a**,**c**,**e**): blue—Day 0; green—Day 10 @ 40 °C; brown—Day 20 @ 40 °C; olive—placebo (formulation without ACTY116); (**a**,**b**) microspheres (F1); (**c**,**d**) phospholipid-based in situ gel (F2); (**e**,**f**) PLGA-based in situ gel (F3).

Microspheres (F1): Initial impurity profiles (Figure 7a,b, Table 6) revealed that following the preparation of microspheres, there was a 17.09% increase in total impurities. Throughout the accelerated stability, the levels of total impurities exhibited a consistent trend, namely a 26.39% increase after 10 days, which escalated to 41.22% after 20 days.

Phospholipid-based in situ gel (F2): Initial impurity profiles (Figure 7c,d, Table 6) demonstrated a time-dependent increase in total impurities within the phospholipid-based in situ gel under 40 °C conditions. Throughout the accelerated stability, the levels of total impurities exhibited a consistent trend, namely a 16.54% increase after 10 days, which escalated to 33.90% after 20 days.

PLGA-based in situ gel (F3): Initial impurity profiles (Figure 7e,f, Table 6) revealed a similar trend of an increase in the total impurities in F3 compared with F1 and F2 but at a noticeably slower pace. The total impurities exhibited a 4.95% increase after 10 days, which escalated to 9.85% after 20 days.

#### 3.4.2. Initial CQA2 for Different Formulations

The other critical quality attributes (CQA2) for different formulations are presented in Table 7. Among the formulations, microspheres (F1) had the highest level of water content, while phospholipid-based in situ gel (F2) had the highest levels of I_A_, I_I_, I_P_, and AV.

### 3.5. Risk Assessment and Control Strategies

#### 3.5.1. Critical Material Attributes (CMAs) for Excipients

The CQAs were significantly influenced by the contributions of critical excipients. By examining the CMAs of the excipients, the objective was to identify CMAs that demonstrated a robust relationship with CQAs, enabling the formulation of effective control strategies. In microspheres (F1), PVA and mannitol did not come into direct contact with the inner aqueous phase of the peptide in W_1_/O/W_2_ (ACTY116 was in W_1_, and PVA and mannitol were in W_2_); therefore, they were not included in the investigation. CMAs for the essential excipients are presented in Table 8.

For Excipients in F1 and F3, the water content, acid value, iodine value, peroxide value, and AV in the excipients were found to be sufficiently low.

CQA2 analysis results in Table 7 reveal that I_A_, I_I_, I_P_, and AV values of the F2 formulation exceed those of the other two formulations. Data in Table 8 demonstrate that soybean phospholipid has the highest levels of water content, I_A_, I_I_, and I_P_, while MCT has the highest AV. These two excipients were critical ingredients in F2, which can explain the high CQAs of F2. Consequently, the control strategy for this formulation encompassed the selection of excipients with superior CMAs (Table 9), and the updated phospholipid-based in situ gel was designated as F5.

#### 3.5.2. CPP Control Strategies

For microspheres (F1), process parameters related to peptide oxidation or hydrolysis, such as high temperature and oxygen exposure, were identified as CPPs. The control strategies focused on lowering the DCM evaporation temperature from 50 °C to 40 °C while ensuring compliance with residual solvent regulations; extending the secondary drying phase of the lyophilization process from 10 h to 20 h to reduce water content; and replacing the air in the vial headspace with nitrogen to maintain the oxygen level at ≤0.1%. The updated microsphere formulation was designated as F4.

For the in situ gel formulations (F2 and F3), an uncomplicated preparation process was employed without harsh conditions such as high temperatures. The most effective strategy was headspace oxygen level control. The control strategy for phospholipid-based in situ gel encompassed CPP control through the replacement of air in the vial headspace with nitrogen to maintain the oxygen level at ≤0.1%, coupled with the selection of excipients with superior CMAs. The updated phospholipid-based in situ gel was designated as F5. The control strategy for PLGA-based in situ gel was the replacement of air in the vial headspace with nitrogen to maintain the oxygen level at ≤0.1%. The updated PLGA-based in situ gel was designated as F6.

The control strategies for the three LAI formulations are summarized in Table 10.

### 3.6. Updated CQAs for Different Formulations

**Figure 8:**
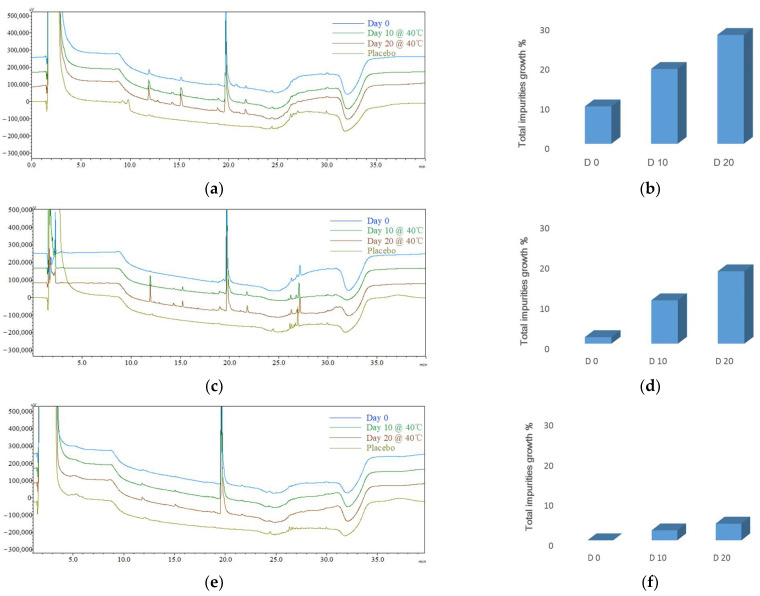
The typical HPLC chromatograms (left: **a**,**c**,**e**; the *X*-axis represents the time, and the *Y*-axis represents the signal intensity) and the impurity profile charts (right: **b**,**d**,**f**) of the updated impurity profile for different formulations. The typical chromatograms (left: **a**,**c**,**e**): blue—Day 0; green—Day 10 @ 40 °C, brown—Day 20 @ 40 °C; olive—placebo (formulation without ACTY116); (**a**,**b**) microspheres (F4); (**c**,**d**) phospholipid-based in situ gel (F5); (**e**,**f**) PLGA-based in situ gel (F6).

The updated impurity profiles (CQA1) for all three formulations demonstrated improvement following the implementation of the control strategies (Figure 8, Table 10). After the completion of microsphere (F4) preparation, a 9.42% increase in total impurities was observed. Subsequently, during the 10-day accelerated storage, total impurities exhibited an 18.85% increase, which further escalated to a 27.43% increase after 20 days. In the case of phospholipid-based in situ gel (F5), there was a 1.62% increase in total impurities following preparation, and during the 10-day accelerated storage, the total impurities displayed a growth of 10.75%, which further increased to 17.95% after 20 days. Surprisingly, for PLGA-based in situ gel (F6), there was no observable increase in total impurities after preparation, and the accelerated stability resulted in a notably sluggish rate of increase under 40 °C conditions: the total impurities increased by 2.48% after 10 days and increased by 4.17% after 20 days.

**Figure 9:**
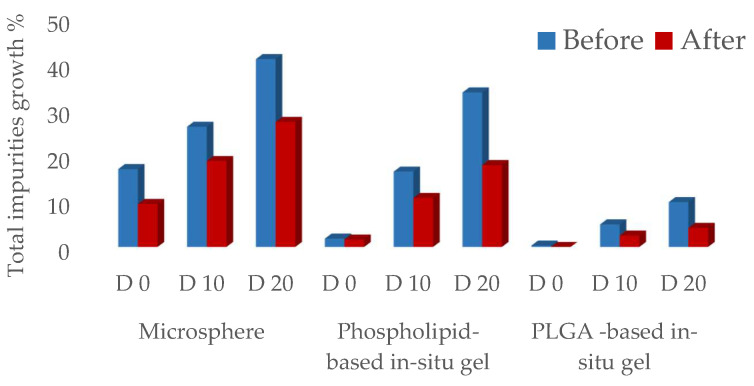
The impurity profile chart of three formulations before and after implementation of control strategies.

The introduction of a comprehensive set of control strategies resulted in notable enhancements of ACTY116 stability in all three formulations. The updated CQAs for LAI formulations with control strategies are listed in Table 10, and the impurity profile charts of the three formulations before and after the implementation of the control strategies are visually represented in Figure 9. Remarkably, owing to the exceptional CMAs of excipients within the PLGA-based in situ gel, the absence of moisture and high temperature during the preparation process, coupled with the CPP control over low levels of headspace oxygen, contributed to the most favorable impurity profile observed in F6.

### 3.7. Pharmacokinetic Studies in Rats

**Figure 10:**
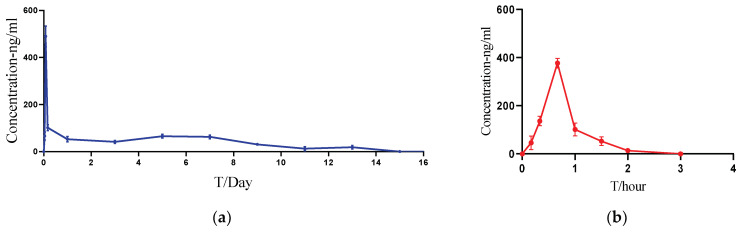
ACTY116 PK profiles: (**a**) ACTY116 LAI formulation (F6); (**b**) ACTY116 solution.

PLGA-based in situ gel (F6) was chosen as the LAI formulation for further pharmacokinetic investigations in rats. Pharmacokinetic parameters (Table 11) and profiles (Figure 10) showed that ACTY116 LAI formulation could significantly extend both the half-life and the mean residence time (MRT) from less than 1 h to over 100 h.

### 3.8. Evaluation of Target Product Profile Achievement

Applying the QbD concept to the formulation design, control strategies were devised and implemented after identifying CQAs, CMAs, and CPPs for each formulation. These strategies resulted in notable CQA improvements for all formulations. When compared to the target product impurity profile, only F6 successfully met the goal (Table 12).

PLGA-based in situ gel exhibited prolonged ACTY116 release and extended residence time compared to the corresponding solution in the PK study. T_last_ values in Table 11 showed that after 2 h following the administration of the solution, ACTY116 was undetectable in the blood, while the LAI formulation maintained a measurable concentration for about 13 days (312 ± 19.2 h). This indicated that the LAI formulation achieved the long-acting goal for at least one week in rats.

## 4. Discussion

The purpose of this study was to utilize the QbD concept to develop a stable ACTY116-loaded long-acting injection. Simultaneously addressing peptide chemical instability and achieving long-acting release presents a substantial challenge, which is the novelty of this research. We chose biodegradable microspheres and in situ gels as the dosage forms for long-acting drug delivery. For hydrophilic peptides, the preparation of PLGA microspheres was challenging, and encapsulation efficiency was low. As demonstrated by the results of microsphere formulations F1 and F4 in this study, the preparation method not only involved a complex process but also led to peptide degradation under high temperatures. In situ gel formation with an uncomplicated preparation process was designed, and a nonaqueous solvent was selected to overcome these challenges. The principle guiding the choice of this nonaqueous solvent is that the sustained-release matrix can dissolve in the solvent, while the peptide cannot. This results in the formation of a peptide suspension in the solvent. Upon administration, the solvent diffuses into surrounding tissues, forming a solid or semisolid drug delivery depot comprising the peptide and sustained-release matrix. This transformed depot exhibits prolonged residence at the injection site, facilitating sustained drug release [40,41].

We conducted our study by following QbD principles. An in-depth comprehension of peptide degradation mechanisms was achieved through forced degradation investigations, elucidating (acid) hydrolysis and oxidation as the primary pathways for the peptide ACTY116 degradation. The quality built into the product was focused on risk assessment, for which the critical material attributes (CMAs) and critical process parameters (CPPs) associated with the critical quality attributes (CQAs) of each formulation were identified, leading to the development of corresponding control strategies. Following the implementation of a series of control strategies, the CQAs for all three formulations improved, and the PLGA-based in situ gel (F6) achieved the target product impurity profile; thus, it was selected as the LAI formulation for subsequent pharmacokinetic studies. PK profiles showed that the ACTY116 LAI formulation could significantly extend the half-life from less than 1 h to over 100 h. The LAI formulation studies and in vivo pharmacokinetic findings presented in this article establish a robust foundation for the use of ACTY116 in more in-depth preclinical investigations and provide a basis for potential future clinical studies.

The number of novel therapeutic peptide approvals by the US Food and Drug Administration (USFDA) is increasing in the market. About 140 peptide drugs are currently in clinical trials, with more than 500 peptides in preclinical trials [42,43]. Significant research efforts are being made to address drawbacks such as poor stability and short half-life. To this end, structural modifications and novel delivery tactics have been developed to boost their ability to reach their targets as fully functional species. The field is being revolutionized with the inclusion of modern strategies such as synthetic nanochaperone [44] and halloysite nanotubes [45]. In conclusion, the field is in need of novel ideas that can help introduce these peptides to the market.

## 5. Conclusions

The ACTY116 structure has 32 amide bonds and 1 guanidine group, which account for its susceptibility to hydrolysis. Additionally, the presence of one phenolic hydroxyl group, five amino groups, and two hydroxyl groups makes it susceptible to oxidation. Therefore, maintaining its stability is a significant challenge. According to the QbD principle, PLGA-based in situ gel as an LAI formulation was designed, and an uncomplicated preparation process was employed, which avoided the need for harsh conditions like high temperatures, high shear mixing, or homogenization. Furthermore, the maintenance of a water- and oxygen-free environment ensures the chemical stability of peptide ACTY116. For other molecules with susceptible functionalities like amide bonds, amino groups, hydroxyl groups, etc., the utilization of PLGA-based in situ gel as a long-acting injectable formulation for stabilizing the API provides valuable insights.

## Figures and Tables

**Table 1:** Gradient elution process.

Time/Min	0	5	6	20	23	28	30	40
A%	90	90	75	50	10	10	90	90
B%	10	10	25	50	90	90	10	10

**Table 2:** Formulation ingredients.

	Microsphere	Phospholipid-Based In Situ Gel	PLGA-Based In Situ Gel
Ingredients	ACTY116 PLGA DCM PVA Mannitol	ACTY116 Soybean phospholipid MCT	ACTY116 PLGA NMP

**Table 3:** Lyophilization process for ACTY116 microspheres.

Temperature/°C	−40	−40	−20	−20	0	25	25
Duration/min	30	180	120	270	180	120	600
Pressure/Pa	—	—	10–16	10–16	10–16	10–16	10–16

**Table 4:** The total impurities under different forced degradation conditions.

Degradation Condition	Control Sample	Thermal Stress	Photolytic Stress	Oxidative Stress	Hydrolytic Stress	Acidic Hydrolytic Stress
Total impurities %	2.2	2.3	2.2	10.1	9.2	34.1

**Table 5:** Characterization of different formulations.

	Particle Size (μm)				EE%	Viscosity (cP)
	D(10)	D(50)	D(90)	D(4,3)		
F1	5.12	16.6	38.3	21.9	29.8	NA
F2	6.48	26.6	104	41.6	NA	142
F3	7.11	28.5	107	44.1	NA	115

NA means not applicable.

**Table 6:** The initial impurity profiles (CQA1) for different formulations.

	F1	F2	F3
Day 0	17.09 ± 1.18%	1.83 ± 0.87%	0.31 ± 0.08%
Day 10	26.39 ± 4.84%	16.54 ± 0.33%	4.95 ± 0.69%
Day 20	41.22 ± 0.48%	33.90 ± 3.52%	9.85 ± 2.74%

**Table 7:** CQA2 for different formulations.

	Water%	I_A_	I_I_	I_P_	AV
Microspheres (F1)	2.37	0	0	0.2	0
Phospholipid-based in situ gel (F2)	0.6	2.9	37.2	2.6	2.1
PLGA-based in situ gel (F3)	0.03	0	0	0	0

**Table 8:** The CMAs of the critical excipients.

		Water%	I_A_	I_I_	I_P_	AV
F1	PLGA	0.01	0	0	0	0
	DCM	0.06	0	0	0	0
F2	Soybean phospholipid	1.4	8.3	105.3	2.8	0.2
	MCT	0.1	0.2	0.9	1.4	0.6
F3	PLGA	0.01	0	0	0	0
	NMP	0.03	0	0	0	0

**Table 9:** Excipients with different CMAs in F2 and F5.

		Water%	I_A_	I_I_	I_P_	AV
Soybean phospholipid	F2	1.4	8.3	105.3	2.8	0.2
	F5	1.1	1.6	93.7	1.3	0.1
MCT	F2	0.1	0.2	0.9	1.4	0.6
	F5	0.1	0.1	0.8	0.3	0.2

**Table 10:** Updated CQAs for LAI formulations with control strategies.

		Microsphere F4	Phospholipid-Based In Situ Gel F5	PLGA-Based In Situ Gel F6
CQA1 (impurity profiles)	Day 0	9.42 ± 1.31%	1.62 ± 0.79%	0.00%
	Day 10	18.85 ± 1.06%	10.75 ± 0.81%	2.48 ± 0.42%
	Day 20	27.43 ± 3.33%	17.95 ± 1.67%	4.17 ± 0.42%
CQA2	Water%	1.45	0.4	0.01
	I_A_	0	0.6	0
	I_I_	0	35.5	0
	I_P_	0	0.7	0
	AV	0	0.6	0
Control strategies	CMAs	-	Selection of excipients with superior CMAs	-
	CPPs	Lowering the evaporation temperature to 40 °C Extending the secondary drying process to 1200 min Oxygen headspace ≤ 0.1%	Oxygen in headspace ≤ 0.1%	Oxygen in headspace ≤ 0.1%

**Table 11:** PK parameters of ACTY116 solution and ACTY116 LAI formulation.

PK Parameters	C_max_ (ng/mL)	AUC_last_ (h·ng/mL)	T_max_ (h)	HL_Lambda_z (h)	MRT_last_ (h)	T_last_ (h)
ACTY116 Solution	377.4 ± 20.3	240.5 ± 29.1	0.67	0.351 ± 0.021	0.796 ± 0.024	2 ± 0
ACTY116 LAI formulation	489.8 ± 44.3	14,385.6 ± 1063.2	1.99	100.4 ± 50.4	117.5 ± 7.2	312 ± 19.2

**Table 12:** Assessment of target product impurity profile achievement.

	The Total Impurities’ Growth		
	During Preparation	10 Days @ 40 °C	20 Days @ 40 °C
Target product impurity profile	≤1.0%	≤3.5%	≤7.0%
Microspheres (F4)	9.42 ± 1.31%	18.85 ± 1.06%	27.43 ± 3.33%
Phospholipid-based in situ gel (F5)	1.62 ± 0.79%	10.75 ± 0.81%	17.95 ± 1.67%
PLGA-based in situ gel (F6)	0	2.48 ± 0.42%	4.17 ± 0.42%

## Data Availability

Data are contained within the article.

## References

[1] Yang H., Liu Y., Lu X.L., Li X.H., Zhang H.G. (2013). Transmembrane transport of the gαq protein carboxyl terminus imitation polypeptide gcip-27. Eur. J. Pharm. Sci..

[2] Wang Y., Lu X.L., Yang H., Li X.H., Zhang H.G. (2011). Effects of polypeptide drug huilixinkang on cardiac hypertrophy and expressions of myosin heavy chain in mice. Chin. Pharm. J..

[3] Lu X.L., Tong Y.F., Liu Y., Xu Y.L., Yang H., Zhang G.Y., Li X.H., Zhang H.G. (2015). Gαq protein carboxyl terminus imitation polypeptide gcip-27 improves cardiac function in chronic heart failure rats. PLoS ONE.

[4] Blessy M.R.D.P., Patel R.D., Prajapati P.N., Agrawal Y.K. (2014). Development of forced degradation and stability indicating studies of drugs—A review. J. Pharm. Anal..

[5] Naazneen S., Sridevi A. (2017). Development of assay method and forced degradation study of valsartan and sacubitril by RP-HPLC in tablet formulation. Int. J. Appl. Pharm..

[6] Jimmidi R. (2023). Synthesis and applications of peptides and peptidomimetics in drug discovery. Eur. J. Org. Chem..

[7] Nugrahadi P.P., Hinrichs W.L., Frijlink H.W., Schöneich C., Avanti C. (2023). Designing formulation strategies for enhanced stability of therapeutic peptides in aqueous solutions: A review. Pharmaceutics.

[8] Shi Y., Lu A., Wang X., Belahadj Z., Wang J., Zhang Q. (2021). A review of existing strategies for designing long-acting parenteral formulations: Focus on underlying mechanisms, and future perspectives. Acta Pharm. Sin. B..

[9] Ahmed N.R., Kulkarni V.V., Pokhrel S., Akram H., Abdelgadir A., Chatterjee A., Khan S. (2022). Comparing the efficacy and safety of obeticholic acid and semaglutide in patients with non-alcoholic fatty liver disease: A systematic review. Cureus.

[10] Knudsen L.B., Lau J. (2019). The discovery and development of liraglutide and semaglutide. Front. Endocrinol..

[11] Citrome L. (2010). Paliperidone palmitate—Review of the efficacy, safety and cost of a new second-generation depot antipsychotic medication. Int. J. Clin. Pract..

[12] Li G., Rui-Guo Z., Yu-Ting Q., Yun-Chun C., Hua-Ning W., Psychosomatic D.O. (2017). Efficacy and Safety of Paliperidone Palmitate or Long-acting Injectable Risperidone in Patients with Schizophrenia. Prog. Mod. Biomed..

[13] Fowler J.E., Flanagan M., Gleason D.M., Klimberg I.W., Gottesman J.E., Sharifi R. (2000). Evaluation of an implant that delivers leuprolide for 1 year for the palliative treatment of prostate cancer. Urology.

[14] Park K., Skidmore S., Hadar J., Garner J., Park H., Otte A., Soh B.K., Yoon G., Yu D., Yun Y. (2019). Injectable, long-acting plga formulations: Analyzing plga and understanding microparticle formation. J. Control. Release.

[15] Lee W.Y., Asadujjaman M., Jee J.P. (2019). Long acting injectable formulations: The state of the arts and challenges of poly (lactic-co-glycolic acid) microsphere, hydrogel, organogel and liquid crystal. J. Pharm. Investig..

[16] O’Brien M.N., Jiang W., Wang Y., Loffredo D.M. (2021). Challenges and opportunities in the development of complex generic long-acting injectable drug products. J. Control. Release.

[17] Zhou J., Walker J., Ackermann R., Olsen K., Hong J.K., Wang Y., Schwendeman S.P. (2020). Effect of manufacturing variables and raw materials on the composition-equivalent plga microspheres for 1-month controlled release of leuprolide. Mol. Pharm..

[18] Park K., Otte A., Sharifi F., Garner J., Skidmore S., Park H., Jhon Y.K., Qin B., Wang Y. (2021). Formulation composition, manufacturing process, and characterization of poly (lactide-co-glycolide) microparticles. J. Control. Release.

[19] Nair H.A., Begum N. (2020). Development and evaluation of a poloxamer- and chitosan-based in situ gel-forming injectable depot. Asian J. Pharm. Clin. Res..

[20] Muddineti O.S., Omri A. (2022). Current trends in plga based long-acting injectable products: The industry perspective. Expert Opin. Drug Deliv..

[21] Somayaji M.R., Das D., Przekwas A. (2016). A new level a type ivivc for the rational design of clinical trials toward regulatory approval of generic polymeric long-acting injectables. Clin. Pharmacokinet..

[22] Butreddy A., Gaddam R.P., Kommineni N., Dudhipala N., Voshavar C. (2021). Plga/pla-based long-acting injectable depot microspheres in clinical use: Production and characterization overview for protein/peptide delivery. Int. J. Mol. Sci..

[23] Silva A.T.C.R., Cardoso B.C.O., e Silva M.E.S.R., Freitas R.F.S., Sousa R.G. (2015). Synthesis, characterization, and study of plga copolymer in vitro degradation. J. Biomater. Nanobiotechnol..

[24] Mir M., Ahmed N., ur Rehman A. (2017). Recent applications of plga based nanostructures in drug delivery. Colloids Surf. B Biointerfaces.

[25] Lim Y.W., Tan W.S., Ho K.L., Mariatulqabtiah A.R., Abu Kasim N.H., Abd Rahman N., Wong T.W., Chee C.F. (2022). Challenges and Complications of Poly(lactic-co-glycolic acid)-Based Long-Acting Drug Product Development. Pharmaceutics.

[26] Hu M., Zhang Y., Xiang N., Zhong Y., Gong T., Zhang Z.R., Fu Y. (2016). Long-Acting Phospholipid Gel of Exenatide for Long-Term Therapy of Type II Diabetes. Pharm. Res..

[27] Nanxi X., Yu Z., Xinyi H.E., Jinjie Z., Xun S., Tao G. (2017). Preparation and pharmacokinetic study of Huperzine A phospholipid in situ-gel. West China J. Pharm. Sci..

[28] Li H., Liu T., Zhu Y., Fu Q., Wu W., Deng J., Shi S. (2017). An in situ-forming phospholipid-based phase transition gel prolongs the duration of local anesthesia for ropivacaine with minimal toxicity. Acta Biomater..

[29] Xu X., Dai Z., Zhang Z., Kou X., You X., Sun H., Zhu H. (2021). Fabrication of oral nanovesicle in-situ gel based on Epigallocatechin gallate phospholipid complex: Application in dental anti-caries. Eur. J. Pharmacol..

[30] Yu L.X., Amidon G., Khan M.A., Hoag S.W., Polli J., Raju G.K., Woodcock J. (2014). Understanding pharmaceutical quality by design. AAPS J..

[31] Devi M.S., Babu G.R., Mulukuri N.S. (2017). A new stability indicating RP-HPLC method for the simultaneous estimation of Diloxanide and Ornidazole in bulk and Pharmaceutical Dosage forms. Res. J. Pharm. Technol..

[32] Lakka NSKuppan C., Srinivas K.S., Yarra R. (2020). Separation and Characterization of New Forced Degradation Products of Dasatinib in Tablet Dosage Formulation Using LC-MS and Stability-Indicating HPLC Methods. Chromatographia.

[33] Rathor S., Sherje A. (2021). Forced degradation studies of tizanidine hydrochloride and development of validated stability-indicating RP-HPLC method. Indian Drugs.

[34] Li Z., Mu H., Larsen S.W., Jensen H., Østergaard J. (2020). Initial leuprolide acetate release from poly (d, l-lactide-co-glycolide) in situ forming implants as studied by ultraviolet–visible imaging. Mol. Pharm..

[35] Ibrahim T.M., El-Megrab N.A., El-Nahas H.M. (2020). Optimization of injectable plga in-situ forming implants of anti-psychotic risperidone via box-behnken design. J. Drug Deliv. Sci. Technol..

[36] Shukr M.H., Ismail S., El-Hossary G.G., El-Shazly A.H. (2021). Design and evaluation of mucoadhesive in situ liposomal gel for sustained ocular delivery of travoprost using two steps factorial design. J. Drug Deliv. Sci. Technol..

[37] (2022). European Pharmacopoeia 11.0.

[38] Doleschall F., KemÉNy Z., Recseg K., Kővári K. (2002). A new analytical method to monitor lipid peroxidation during bleaching. Eur. J. Lipid Sci. Technol..

[39] Lai-Lin Z., Qing-He Z., Jie-Sheng Z., Shu-Li W. (2015). Effects of storage conditions on oil-making quality of sesame seeds. J. Henan Univ. Technol..

[40] Vigani B., Rossi S., Sandri G., Bonferoni M.C., Caramella C.M., Ferrari F. (2020). Recent advances in the development of in situ gelling drug delivery systems for non-parenteral administration routes. Pharmaceutics.

[41] Ibrahim T.M., El-Megrab N.A., El-Nahas H.M. (2021). An overview of plga in-situ forming implants based on solvent exchange technique: Effect of formulation components and characterization. Pharm. Dev. Technol..

[42] Al Shaer D., Al Musaimi O., Albericio F., de la Torre B.G. (2022). 2021 FDA TIDES (Peptides and Oligonucleotides) Harvest. Pharmaceuticals.

[43] Lau J.L., Dunn M.K. (2018). Therapeutic peptides: Historical perspectives, current development trends, and future directions. Bioorg. Med. Chem..

[44] Park I.S., Kim S., Yim Y., Park G., Choi J., Won C., Min D.H. (2022). Multifunctional synthetic nano-chaperone for peptide folding and intracellular delivery. Nat. Commun..

[45] Massaro M., Licandro E., Cauteruccio S., Lazzara G., Liotta L.F., Notarbartolo M., Raymo F.M., Sánchez-Espejo R., Viseras-Iborra C., Riela S. (2022). Nanocarrier based on halloysite and fluorescent probe for intracellular delivery of peptide nucleic acids. J. Colloid Interface Sci..

